# Effects of diet-induced weight loss and Roux-en-Y gastric bypass on the glycemic and gut hormones profile in patients with severe obesity and diabetes

**DOI:** 10.1016/j.clinsp.2025.100736

**Published:** 2025-09-02

**Authors:** Andréa de Fátima Cristino Bastos Crespo, Leila Antoangelo, Priscila Costa Estabile, Roberto de Cleva, Marco Aurelio Santo

**Affiliations:** Hospital das Clinicas (HC), Universidade de Sao Paulo (USP), São Paulo, SP, Brazil

**Keywords:** Diabetes mellitus, Incretins, Very low calorie diet, Glucagon-like peptide-1

## Abstract

**Objectives:**

The mechanisms following glycemic control after RYGB are still not fully elucidated. The objective of the present study was to compare the effects of similar weight loss induced by a Very Low Calorie Diet (VLCD) and by Roux-en-Y Gastric Bypass (RYGB) on the glycemic and gut hormone profile in patients with severe obesity and T2DM.

**Methods:**

A prospective controlled observational study with 10 patients evaluated before (pre-0), after 10 % weight loss induced by VLCD (pre-1), and after 10 % weight loss induced by RYGB (post). Oral Glucose Tolerance Test (OGTT) was performed in pre-0 and pre-1. In all periods, glycemic (glycemia, insulin) and gut hormone profile (Ghrelin, GIP and GLP1) were evaluated in fasting condition (T0) and 30-, 60-, 90- and 120-minutes after a standard meal (Nutren 1.5®).

**Results:**

After 10 % weight loss induced by VLCD 5 patients had remission of T2DM in OGTT. There was a significant increase in ghrelin levels after 10 % weight loss with VLCD (pre-1; *p* < 0.001). There was no difference in GIP levels. There was a significant increase in GLP1 levels between pre-0 and pre-1 (*p* < 0.004) at T0 and between pre-1 and post at all times after a standard meal (*p* = 0.01; *p* = 0.002; *p* = 0.002; *p* = 0.01; *p* = 0.04).

**Conclusions:**

50 % of patients had glycemic control after diet-induced weight loss. After RYGB, all patients had improved glycemic control. The increase of GLP1 after weight loss induced by metabolic surgery is responsible for glycemic control.

## Highlights

• Question – Are early changes in glycemic and gut hormones similar after weight loss induced by VLCD and bariatric surgery?.
• Findings – 50 % of patients had glycemic control after diet-induced weight loss. All patients had improved glycemic control after RYGB. There was an increase of GLP1 after weight loss by diet only in fasting and at all times after surgery.
• Meaning – 50 % of patients had glycemic control after VLCD and 100 % after RYGB.

## Introduction

Bariatric surgery is considered the most successful treatment for Type 2 Diabetes (T2DM) in patients with severe obesity due to better glycemic control in comparison with clinical treatment.^1^^,^^2^

Patients with obesity and T2DM undergoing Roux-en-y Gastric Bypass (RYGB) had an early improvement in glucose levels and insulin resistance, together with an increase in Glucagon-Like Peptide-1 (GLP-1) and Glucose-dependent Insulinotropic Polypeptide (GIP) a few days after surgery.^3^, ^4^, ^5^

The role of proximal bowel exclusion and distal bowel stimulation of L cells are important since the earlier intestinal transit time for nutrients to the distal ileum contributes to an improvement of the glycemic profile after RYGB.^6^^,^^7^, ^8^, ^9^

In spite of this knowledge, a recent study involving patients with severe obesity and T2DM, comparing equal weight loss induced by diet and RYGB, concluded that similar metabolic benefits were observed in both groups.^10^^,^^11^

The mechanisms involved in the early glycemic improvement after RYGB are still not fully elucidated. The incretin effects may depend to a greater or lesser extent on weight loss, nutritional changes, or the gut hormone effects triggered by the procedure.^4^^,^^12^, ^13^, ^14^, ^15^

The hypothesis was that initial glycemic control depends on weight loss, with an additional effect on gut hormone after RYGB.^2^^,^^16^^,^^17^

The aim of this study was to compare the glycemic and gut hormone profiles in patients with severe obesity and T2DM after similar weight loss induced by diet and by RYGB.^13^^,^^14^

## Methods

A prospective controlled observational study with patients recruited from the Metabolic and Bariatric Unit, Hospital das Clínicas da Faculdade de Medicina da Universidade de São Paulo. Ten consecutive patients with severe obesity (BMI > 35 kg/m^2^) and T2DM were evaluated between 2016 to 2018. All participants had 18 to 60 years, BMI > 35 kg/m^2^, T2DM diagnosed < 10-years, a C-peptide level > 3 ng/mL and HbA1c < 12.5 %. Patients under insulin or GLP 1 agonist therapy, use of oral or injected steroids for more than 14 consecutive days in the last 3-months, and with hepatitis B, C and HIV were excluded.

This study was performed according to the ethical recommendations of the Declaration of Helsinki and approved by the Research Ethics Committee of the referred Hospital under registration CAAE: 39,590,814.50000.0068. All participants provided written informed consent before participating in this study.

### Study protocol

**Fig. 1:**
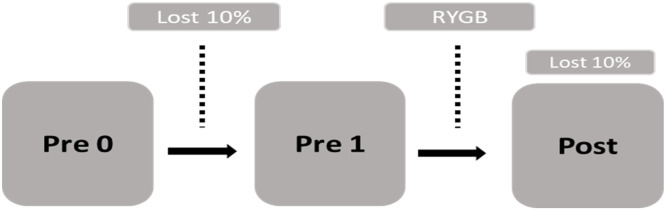
Schematic illustration of protocol.

The selected patients were evaluated before surgery (pre-0), after 8 %‒10 % weight loss (pre-1) induced by a Very Low-Calorie Diet (VLCD), and after 8 %‒10 % weight loss induced by RYGB (post) (Fig. 1). All patients were submitted to the Oral Glucose Tolerance Test (OGTT) in pre-0 and pre-1. All patients underwent gut hormone profile (insulin, ghrelin, GIP and GLP1) evaluation at times T0 (fasting) and T30, T60, T90 and T120 min after ingestion of a standard meal of 300 kcal (Nutren 1.5®).

The insulin resistance was determined according to the formula below: HOMA−IR=(fastinginsulin(μUI/mL)×fastingglucose(mg/dL)×(0.0555)/22.5(50).

The gut hormone profile was collected in a tube containing EDTA anticoagulant, centrifuged at 4500 rpm at 4 °C for 15 minutes for plasma separation and then was stored at −80 °CA. Plasma ghrelin, GIP, GLP1 were measured using multiplex immunoassays (Luminex® xMAP).

The surgical technique was Roux-en-Y Gastric Bypass (RYGB), with a gastric pouch of 30 to 50 mL, 70 to 80 cm of biliopancreatic limb, and 100 to 120 cm of alimentary limb.

### Statistical analysis

Continuous variables were expressed as mean, median, standard deviation, and quartiles. The Non-parametric ANOVA test for repeated measures was used to compare three times or more. The comparison between the two times was performed using the Mann-Whitney test paired with Benjamini-Hochberg significance correction. The Area Under the Curve (AUC) was calculated using the trapezoid method. The profile graphs were constructed using the median and interquartile range and also by the mean and standard deviation, used to graphically express the measures that vary over time. Boxplot plots were used to illustrate continuous measures that do not vary with time. The significance level adopted in the tests was 5 % (*p* < 0.05) using a two-tailed hypothesis. The R software version 3.6 was used to perform all analyses.^1^

## Results

Demographic characteristics are shown in Table 1. Ten patients (50.6 ± 9.5 years) were included (5 female) with a BMI of 43.5 ± 3.6 Kg/m^2^ and a mean time of T2DM of 4.7 ± 1.3 years. In pre-0 glycemia was 147.5 ± 50.4 mg/dL, HbA1c 8.0 % ± 2.0 % and C-peptide 7.2 ± 2.8 ng/mL.

**Table 1:** Results of demographic, anthropometric and laboratory data in Pre-0.

Demographic data	
Sex	5 Male
	5 Female
Age	50.6 ± 9.5 years
Weight	120.7 ± 14.4 kg
Height	1.68 ± 0.13 m
BMI	43.5 ± 3.6 kg/m^2^
Diabetes diagnosis	4.7 ± 1.3 years
Glycemia	147.5 ± 50.3 mg/dL
HbAIC	8.0 ± 2.0 %
Peptide-C	7.2 ± 2.8 mg/mL

BMI, Body Mass Index; HbAIC, Glycated Hemoglobin.

The average weight loss after VLCD was 11.3 ± 1.6 Kg (8.59 %) in 26.5 ± 9.0 days, and 50 % of the patients had glycemic control in OGTT.

Weight equivalent reductions after RYGB were 11.4 ± 3.9 Kg (9.45 %) in 49.5 ± 13.5 days and 100 % of the patients had glycemic control.

### Glycemic profile

**Fig. 2:**
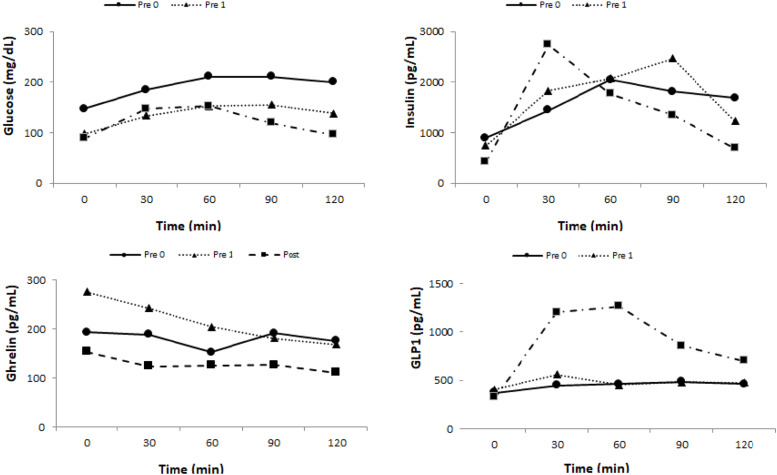
The profile graphic of descriptive values of Glucose, Insulin, Ghrelin and GLP1 in pre-0, pr-1 and post-surgery.

The results of the glycemic profile are in Table 2 and Fig. 2.

**Table 2:** Descriptiveanalysisof Glycemia and Insulin.

	Glycemia (mg/dL)			Insulin (pg/mL)		
**Time**	**pre-0**	**pre-1**	**post**	**pre-0**	**pre-1**	**post**
0	147.5 ± 50.2	97.5 ± 14.8^a^	89 ± 9.4^b^	1128.1 ± 68.2	841.5 ± 399.7	474.6 ± 91.3^c^
30	184.8 ± 66.1	133.7 ± 27.5	147 ± 16.8	2532.1 ± 2641.4	2025.9 ± 1095	3085.5 ± 2122.5
60	210.8 ± 75.5	152.3 ± 36.3	152.3 ± 38.1	3099.1 ± 3625	2716.9 ± 2288.8	2957.6 ± 2418.2
90	211.5 ± 76.7	154.9 ± 38.5	119 ± 31.6	3894.3 ± 4770.1	2221.4 ± 1194.4	1927.6 ± 1727.7
120	200.4 ± 73	138.4 ± 38.2^a^	95.7 ± 18^b^^,^^c^	2842.7 ± 2787.6	1547.1 ± 906.8	1123.1 ± 929.2^a^

Mann-Whitney test paired. Results are expressed as mean + SD.

Where: pre-0 = Pre-surgical 0; pre-1 = After 10 % weight loss by VLCD; post = After 10 % weight loss by RYGB.

a pre-0 × pre-1, *p* < 0.01.

b pre-0 × post, *p* < 0.01.

c pre-1 × post, *p* < 0.01.

### Glycemia

There was a significant reduction of fasting glycemia at T0 between pre-0 and pre-1 (*p* = 0.006) and pre-0 and post (*p* = 0.002). There was also a significant reduction of T 120 glycemia between pre-0 and pre-1 (*p* = 0.027), pre-1 and post (*p* = 0.004), and pre-0 and post (*p* = 0.002).

### Insulin

The basal insulin level had a significant reduction between pre-0 and post (*p* = 0.006) and pre-1 and post (*p* = 0.006). At T 120 min there was also a significant reduction between pre-0 and post (*p* = 0.037) (Table 2 and Fig. 2).

### Homa IR

There was a significant reduction of Homa IR between pre-0 and post (*p* = 0.006) and between pre-1 and post (*p* = 0.018).

### Gut hormone profile

#### Ghrelin

There was an increase in Ghrelin levels between pre-0 and pre-1 (*p* = 0.020) and a significant reduction between pre-1 and post (*p* = 0.014) at T0. At T120, there was a reduction between pre-1 and post (*p* = 0.006) (Table 3 and Fig. 2).

**Table 3:** Descriptiveanalysisof Ghrelin, GIP and GLP1.

Ghrelin	Pre-O			Pre-1			Post		
T(MIN)	MD	Q1	Q3	MD	Q1	Q3	MD	Q1	Q3
0	193.4	137.1	623.8	275.9^a^	205.4	1250	154.2^b^	102.4	310.8
30	189	129.5	505	243.1	161	1222.9	123.7	115	272.3
60	153.3	124.7	564.3	204.8	168.6	1146.3	125.6	106.3	230.2
90	192.2	136.2	494	181.6	152.6	1170.7	126.6	100.9	180.1
120	175.9	124.2	391.8	168.9	139.5	996.1	110.8^b^	98.66	196.1
**GIP**		**Pre-0**			**Pre-1**		**Post**		
0	382.6	294.9	569.8	370.8	234.5	460.7	284.3	226.2	348.6
30	1111	1007.3	1360.8	1058	606.5	1250.5	1459	1100.8	1592.5
60	1084	973.2	1371.8	997.5	705.5	1275	1154	784.9	1772.8
90	1226	782	1373.8	1127	820.8	1391.3	876.6	605.1	1284.8
120	981.4	751	1408.5	893.3	548.7	1116	749.5	488.2	957.6
**GLP1**		**Pre-0**			**Pre-1**			**Post**	
0	371	250.3	465.8	406.6^a^	327	453.8	325.0^b^	231.8	387.4
30	446	285.3	673.7	559	412.5	745.8	1204.5^a^^,^^b^	1051	1954.5
60	463.6	312.1	545.5	453.4	394	787.6	1262.0^a^^,^^b^	653	2212.8
90	485.2	310.9	597.9	480.8	379.1	921.9	857.7^a^^,^^b^	532.8	1393.1
120	463.3	315.8	542.4	477.8	341	763.5	701.6^a^^,^^b^	427	1145.3

Where: pre-0 = pre-surgical 0; pre-1 = after 10 % weight loss by VLCD; post = after 10 % weight loss by RYGB.

a pre-0 × pre-1, *p* < 0.01.

b pre-0 × post, *p* < 0.01; *** pre-1 × post, *p* < 0.01.

### GIP

There were no significant variations in GIP levels at all times studied (Table 3 and Fig. 2).

### GLP1

There was a significant increase in GLP1 level between pre-0 and pre-1 only at T0 (*p* = 0.044). There was a significant increase between pre-0 and post (T30 *p* = 0.002; T60 *p* = 0.002; T90 *p* = 0.01 and T120 *p* = 0.01) and between pre-1 and post at all times (T0 *p* = 0.01; T30 *p* = 0.002; T60 *p* = 0.002; T90 *p* = 0.01 and T120 *p* = 0.04) (Table 3 and Fig. 2).

## Discussion

The present study compared in the same group of patients the early effects of 10 % weight loss induced by a VLCD to an equivalent weight loss after RYGB. 50 % of the patients had an improvement in the glycemic profile after the diet, with normalization of the OGTT. After RYGB, all patients showed an improvement in the glycemic profile.^17^

There was a significant improvement in fasting glycemia after 10 % weight loss by diet.^3^^,^^14^^,^^15^

These observations may be partially explained by the improvement of hepatic insulin sensitivity after severe calorie restriction, while peripheral insulin sensitivity improves late in response to postoperative weight loss. With early glycemic control, there is a reduction in fasting glucose and insulin levels and, consequently, a decrease in insulin resistance.^18^

The VLCD diet determines an immediate metabolic improvement, reducing the glucotoxicity and lipotoxicity present in T2DM in approximately 2-weeks. An improvement in the first phase of insulin secretion and an increase in hepatic sensitivity to insulin were observed, but without changes in peripheral insulin resistance.^12^

Ballantyne et al. (2006) studied insulin resistance 3-months after adjustable gastric banding in comparison to RYGB, observing a greater reduction in HOMA-IR levels after RYGB. Caloric restriction also promotes an important improvement in insulin resistance, but after RYGB, there was an additional gut hormone effect caused by the alteration of the passage of food by the diversion of intestinal transit.^4^^,^^19^

The effects of weight loss in a group of individuals with caloric restriction in comparison with another group submitted to RYGB showed that similar weight reduction induced by diet or surgery promotes considerable improvement in β-cell function due to increased glucose sensitivity in both groups, without difference between them.^10^ However, weight loss after the surgical procedure is more intense and effective than diet therapy alone for patients with severe obesity and T2DM.^20^^,^^21^ The results of this study found a significant improvement in T2DM control after RYGB and demonstrate the intrinsic effects of the anatomical changes in the digestive tract generated by the surgery, which add to the effects of weight loss on glucose metabolism.^22^

The ghrelin had increased secretion after 10 % weight loss induced by VLCD in fasting. In contrast, RYGB determined an early important suppression of its secretion.^12^^,^^14^^,^^23^

In the present study, the GIP levels were not different in all studied periods. GIP has an attenuated incretin effect on insulin secretion in individuals with T2DM and can be restored with a reduction in blood glucose after RYGB.^15^^,^^20^^,^^24^^,^^25^

There was no significant increase in GLP1 secretion after diet-induced weight loss.

After RYGB, the significant increase in GLP1 levels between T30 and T60, with better glycemic control, and increased insulin levels, demonstrates the positive effects of surgery. The authors may consider the beneficial addictive effect of weight loss before and after RYGB on glycemic control.^5^, ^17^ Another study conducted by the group evaluated glycemic control with oral and gastrostomy stimulation after RYGB in patients with obesity and T2DM.^3^^,^^16^ The improvement in incretin response with an increase in GLP1 was observed only through the oral route.^3^^,^^26^

The present results obtained after two interventions contributed to reinforcing the metabolic heterogeneity of patients with obesity and T2DM, probably suggesting different treatments according to objective measurement of metabolic impairment or predictors of success for T2DM.^27^

The main limitations of this study were the small number of patients included and the additive effects of weight loss before RYGB on glycemic control.

The impact of metabolic surgery in comparison with drug therapy on the maintenance of remission of TDM2 was evaluated 3-years later. The magnitude of metabolic surgery was stronger than drug therapy and lifestyle change in the remission of T2DM, including individuals with Grade 1 obesity.^17^^,^^28^

Finally, it can be inferred from the results that diet-induced weight loss allows specific benefits in the glycemic profile, which are highly potentiated with surgical treatment.^17^^,^^25^

## Conclusion

50 % of patients had glycemic control after diet-induced weight loss. After RYGB, all patients had improved glycemic control. The increase of GLP1 after weight loss induced by metabolic surgery is responsible for glycemic control.

## Declaration of competing interest

The authors declare no conflicts of interest.

## References

[1] Lingvay I., Guth E., Islam A., Livingston E. (2013). Rapid improvement in diabetes after gastric bypass surgery: is it the diet or surgery?. Diabetes Care.

[2] de Cleva R., Kawamoto F., Borges G., Caproni P., Cassenote A.J.F., Santo M.A. (2021). C-peptide level as predictor of type 2 diabetes remission and body composition changes in non-diabetic and diabetic patients after Roux-en-Y gastric bypass. Clinics (Sao Paulo).

[3] Fernandes G., Santo M.A., Crespo A.F.C.B., Biancardi G.B., Mota F.C., Antonangelo L. (2019). Early glycemic control and incretin improvement after gastric bypass: the role of oral and gastrostomy route. Surg Obes Relat Dis.

[4] Ballantyne G.H., Farkas D., Laker S., Wasielewski A. (2006). Short-term changes in insulin resistance following weight loss surgery for morbid obesity: laparoscopic adjustable gastric banding versus laparoscopic Roux-en-Y gastric bypass. Obes Surg.

[5] Umeda L.M., Silva E.A., Carneiro G., Arasaki C.H., Geloneze B., Zanella M.T. (2011). Early improvement in glycemic control after bariatric surgery and its relationships with insulin, GLP-1, and glucagon secretion in type 2 diabetic patients. Obes Surg.

[6] Estabile P.C., Almeida M.C., Campagnoli E.B., Santo M.A., Rodrigues M.R.S., Milléo F.Q. (2022).

[7] Pournaras D.J., Nygren J., Hagström-Toft E., Arner P., Le Roux C.W., Thorell A. (2016). Improved glucose metabolism after gastric bypass: evolution of the paradigm. Surg Obes Relat Dis.

[8] Rubino F., Forgione A., Cummings D.E., Vix M., Gnuli D., Mingrone G. (2006). The mechanism of diabetes control after gastrointestinal bypass surgery reveals a role of the proximal small intestine in the pathophysiology of type 2 diabetes. Ann Surg.

[9] Buchwald H., Estok R., Fahrbach K., Banel D., Jensen M.D., Pories W.J. (2009). Weight and type 2 diabetes after bariatric surgery: systematic review and meta-analysis. Am J Med.

[10] Yoshino M., Kayser B.D., Yoshino J., Stein R.I., Reeds D., Eagon J.C. (2020). Effects of diet versus gastric bypass on metabolic function in diabetes. N Engl J Med.

[11] Badman M.K., Flier J.S. (2005). The gut and energy balance: visceral allies in the obesity wars. Science.

[12] Kamvissi-Lorenz V., Raffaelli M., Bornstein S., Mingrone G. (2017). Role of the gut on glucose homeostasis: lesson learned from metabolic surgery. Curr Atheroscler Rep.

[13] Isbell J.M., Tamboli R.A., Hansen E.N., Saliba J., Dunn J.P., Phillips S.E. (2010). The importance of caloric restriction in the early improvements in insulin sensitivity after Roux-en-Y gastric bypass surgery. Diabetes Care.

[14] Laferrère B. (2011). Diabetes remission after bariatric surgery: is it just the incretins?. Int J Obes (Lond).

[15] Laferrère B. (2016). Bariatric surgery and obesity: influence on the incretins. Int J Obes Suppl.

[16] Kashyap S.R., Daud S., Kelly K.R., Gastaldelli A., Win H., Brethauer S. (2010). Acute effects of gastric bypass versus gastric restrictive surgery on beta-cell function and insulinotropic hormones in severely obese patients with type 2 diabetes. Int J Obes (Lond).

[17] Laferrère B., Teixeira J., McGinty J., Tran H., Egger J.R., Colarusso A. (2008). Effect of weight loss by gastric bypass surgery versus hypocaloric diet on glucose and incretin levels in patients with type 2 diabetes. J Clin Endocrinol Metab.

[18] Holst J.J., Madsbad S., Bojsen-Møller K.N., Svane M.S., Jørgensen N.B., Dirksen C. (2018). Mechanisms in bariatric surgery: gut hormones, diabetes resolution, and weight loss. Surg Obes Relat Dis.

[19] Bose M., Teixeira J., Olivan B., Bawa B., Arias S., Machineni S. (2010). Weight loss and incretin responsiveness improve glucose control independently after gastric bypass surgery. J Diabetes.

[20] Dirksen C., Jørgensen N.B., Bojsen-Møller K.N., Jacobsen S.H., Hansen D.L., Worm D. (2012). Mechanisms of improved glycaemic control after Roux-en-Y gastric bypass. Diabetologia.

[21] Berggren J., Lindqvist A., Hedenbro J., Groop L., Wierup N. (2017). Roux-en-Y gastric bypass versus calorie restriction: support for surgery per se as the direct contributor to altered responses of insulin and incretins to a mixed meal. Surg Obes Relat Dis.

[22] Foo J., Krebs J., Hayes M.T., Bell D., Macartney-Coxson D., Croft T. (2011). Studies in insulin resistance following a very low calorie diet and/or gastric bypass surgery. Obes Surg.

[23] Laferrère B. (2009). Effect of gastric bypass surgery on the incretins. Diabetes Metab.

[24] Pournaras D.J., Nygren J., Hagström-Toft E., Arner P., Le Roux C.W., Thorell A. (2016). Improved glucose metabolism after gastric bypass: evolution of the paradigm. Surg Obes Relat Dis.

[25] Xie C., Jones K.L., Rayner C.K., Wu T. (2020). Enteroendocrine hormone secretion and metabolic control: importance of the region of the gut stimulation. Pharmaceutics.

[26] Santo M.A., Riccioppo D., Pajecki D., Kawamoto F., de Cleva R., Antonangelo L. (2016). Weight regain after gastric bypass: influence of gut hormones. Obes Surg.

[27] Rubino F., Nathan D.M., Eckel R.H., Schauer P.R., Alberti K.G.M.M., Zimm P.Z. (2016). Metabolic surgery in the treatment algorithm for type 2 Diabetes: a joint statement by International Diabetes Organizations. Diabetes Care.

[28] Kirwan J.P., Courcoulas A.P., Cummings D.E., Goldfine A.B., Kashyap S.R., Simonson D.C. (2022). Diabetes remission in the alliance of Randomized trials of medicine versus metabolic surgery in type 2 Diabetes (ARMMS-T2D). Diabetes Care.

